# Twenty-Year Trends in Outcomes for Older Adults With Acute Myocardial Infarction in the United States

**DOI:** 10.1001/jamanetworkopen.2019.1938

**Published:** 2019-03-15

**Authors:** Harlan M. Krumholz, Sharon-Lise T. Normand, Yun Wang

**Affiliations:** 1 Section of Cardiovascular Medicine, Department of Internal Medicine, Yale School of Medicine, New Haven, Connecticut; 2 Department of Health Policy and Management, Yale School of Public Health, New Haven, Connecticut; 3 Center for Outcomes Research and Evaluation, Yale–New Haven Hospital, New Haven, Connecticut; 4 Department of Health Care Policy, Harvard Medical School, Harvard T.H. Chan School of Public Health, Boston, Massachusetts; 5 Department of Biostatistics, Harvard T.H. Chan School of Public Health, Boston, Massachusetts

## Abstract

**Importance:**

Medicare and other organizations have focused on improving quality of care for patients with acute myocardial infarction (AMI) over the last 2 decades. However, there is no comprehensive perspective on the evolution of outcomes for AMI during that period, and it is unknown whether temporal changes varied by patient subgroup, hospital, or county.

**Objective:**

To provide a comprehensive evaluation of national trends in inpatient outcomes and costs of AMI during this period.

**Design, Setting, and Participants:**

This cohort study included analysis of data from a sample of 4 367 485 Medicare fee-for-service beneficiaries aged 65 years or older from January 1, 1995, through December 31, 2014, across 5680 hospitals in the United States. Analyses were conducted from January 15 to June 5, 2018.

**Main Outcomes and Measures:**

Thirty-day all-cause mortality at the patient, hospital, and county levels. Additional outcomes included 30-day all-cause readmissions; 1-year recurrent AMI; in-hospital mortality; length of hospital stay; 2014 Consumer Price Index–adjusted median Medicare inpatient payment per AMI discharge; and rates of catheterization, percutaneous coronary intervention, and coronary artery bypass graft surgery.

**Results:**

The cohort included 4 367 485 Medicare fee-for-service patients aged 65 years or older hospitalized for AMI during the study period. Between 1995 and 2014, the mean (SD) age of patients increased from 76.9 (7.2) to 78.2 (8.7) years, the percentage of female patients declined from 49.5% to 46.1%, the percentage of white patients declined from 91.0% to 86.2%, and the percentage of black patients increased from 5.9% to 8.0%. There were declines in AMI hospitalizations (914 to 566 per 100 000 beneficiary-years); 30-day mortality (20.0% to 12.4%; difference, 7.6 percentage points; 95% CI, 7.3-7.8 percentage points); 30-day all-cause readmissions (21.0% to 15.3%; difference, 5.7 percentage points; 95% CI, 5.4-6.0 percentage points); and 1-year recurrent AMI (7.1% to 5.1%; difference, 2.0 percentage points; 95% CI, 1.8-2.2 percentage points). There were increases in the 2014 Consumer Price Index–adjusted median (interquartile range) Medicare inpatient payment per AMI discharge ($9282 [$6969-$12 173] to $11 031 [$8099-$16 861]); 30-day inpatient catheterization (44.2% to 59.9%; difference, 15.7 percentage points; 95% CI, 15.4-16.0 percentage points); and inpatient percutaneous coronary intervention (18.8% to 43.3%; difference, 24.5 percentage points; 95% CI, 24.2-24.7 percentage points). Coronary artery bypass graft surgery rates decreased from 14.4% to 10.2% (difference, 4.2 percentage points; 95% CI, 3.9-4.3 percentage points). There was heterogeneity by hospital and county in the mortality changes over time.

**Conclusions and Relevance:**

This study shows marked improvements in short-term mortality and readmissions, with an increase in in-hospital procedures and payments, for the increasingly smaller number of Medicare beneficiaries with AMI.

## Key Points

**Question:**

What are the patterns of hospitalizations, treatments, and outcomes for older patients with acute myocardial infarction (AMI) over a recent 20-year period in the United States?

**Findings:**

In this cohort study of data from more than 4.3 million Medicare fee-for-service beneficiaries aged 65 years and older discharged with AMI, declines were found in AMI hospitalizations (914 to 566 per 100 000 beneficiary-years), 30-day mortality (20.0% to 12.4%), and 30-day all-cause readmissions (21.0% to 15.3%). There were increases in the 2014 Consumer Price Index–adjusted median Medicare inpatient payment per AMI discharge ($9282 to $11 031) and 30-day inpatient catheterization (44.2% to 59.9%).

**Meaning:**

The last 2 decades were marked by large changes in the number of people hospitalized with AMI—and marked improvements in the short- and long-term outcomes along with increases in cost per hospitalization and number of procedures.

## Teaser

This cohort study reports patterns of hospitalizations, treatments, and outcomes for US Medicare fee-for-service beneficiaries aged 65 years or older with acute myocardial infarction from 1995 to 2014.

## Introduction

Acute myocardial infarction (AMI) is undergoing a transition. For the last 2 decades, Medicare has focused on improving quality of care for patients with AMI, starting with the Cooperative Cardiovascular Project.^1^ Studies^2,3,4,5,6,7,8,9,10,11^ have reported improvements in quality and reductions in AMI hospitalizations and risk of short-term mortality during various periods within this time span. However, there is no comprehensive perspective on the evolution of outcomes for AMI across the last 2 decades, including cost, length of stay, treatments, and rehospitalizations. It is also unknown whether changes over time varied by patient subgroup, hospital, or county.

Accordingly, we sought to provide a comprehensive view of treatments and outcomes for patients with AMI over a recent 20-year period in the United States. We used Medicare fee-for-service data to characterize changes in 30-day mortality at the patient, hospital, and county levels and examined trends in the rates of 30-day all-cause readmissions and 1-year recurrent AMI. Finally, we assessed the changes in 3 common in-hospital AMI-specific surgical procedures (catheterization, percutaneous coronary intervention [PCI], and coronary artery bypass graft [CABG]) within 30 days from the date of an AMI index admission.

## Methods

### Study Population

This was a cohort study. The Yale University institutional review board reviewed the study protocol and granted a waiver of informed consent for the use of this deidentified database. We used Medicare enrollment data from the Centers for Medicare & Medicaid Services (CMS) to identify individuals in the Medicare population aged 65 years or older enrolled in the fee-for-service program for at least 1 month between January 1995 and December 2014. We then linked the fee-for-service population data to the Medicare inpatient claims data to identify patients discharged from an acute-care nonfederal hospital from January 1, 1995, through December 31, 2014, with a principal discharge diagnosis of AMI as determined using *International Classification of Diseases, Ninth Revision, Clinical Modification* diagnostic codes 410.xx (with the exception of 410.x2). The linked data include patient demographic characteristics, principal and secondary diagnosis codes, and procedure codes. If a patient had more than 1 AMI hospitalization during the entire study period, we selected the first AMI hospitalization, the index admission, and excluded subsequent hospitalizations (n = 548 546). To ensure that patients did not have an AMI in 1995, we also excluded patients who were hospitalized with an AMI in 1994. The study followed the guidelines for cohort studies described in the Strengthening the Reporting of Observational Studies in Epidemiology (STROBE) reporting guideline for observational studies.^12^

### Patient, Hospital, and County Characteristics

Patient demographic information included age, sex, race (white, black, or other), and dual eligibility status for Medicaid and Medicare. We identified clinical comorbidities that represent additional coexisting illnesses with the method used by CMS to profile hospital 30-day mortality measures for AMI.^13,14^ We determined comorbidities for the index hospitalization from secondary diagnosis codes as well as the principal and secondary diagnosis codes from all hospitalizations for up to 12 months before the index hospitalization. Because the maximum number of diagnosis codes in Medicare data increased from 10 to 25 in 2011,^15^ we restricted the 2011 to 2014 data to the first 10 diagnosis codes to align with the 1995 to 2010 data. Hospital characteristics included hospital structure, patient population, and in-hospital care information. Information about hospital structure was obtained from the 2013 American Hospital Association Surveys, including ownership status, geographic location (urban vs rural), teaching status, Medicare and Medicaid volume, nurse-to-patient ratio, safety-net hospital status (yes vs no), and hospital capacity to provide CABG surgery. Information about in-hospital care included rates of AMI transfer in and transfer out, rates of in-hospital PCI and CABG procedures, and discharge disposition (rates of discharge to home for self-care, to home with home health care, and to skilled nursing home facilities). Information about the patient population and in-hospital care was drawn from CMS inpatient all-cause claims data.

County characteristics included Consumer Price Index–adjusted median income, proportion of people aged 65 years or older, people below the national poverty level (eTable in the Supplement), and a binary variable to indicate whether a county belongs to a health priority area as defined in one of our previous studies.^16^ The priority area has had a persistently high all-cause mortality over time.

### Outcomes

Our primary outcome was 30-day all-cause mortality, defined as death within 30 days from the date of admission for an index AMI hospitalization, regardless of cause. We evaluated mortality at the individual patient level, the hospital level, and the county level. Additional outcomes included (1) 30-day all-cause readmissions, defined as an unplanned rehospitalization within 30 days from the index AMI discharge among individuals who survived to discharge; (2) 1-year recurrent AMI, defined as a recurrent AMI hospitalization within 1 year from the index AMI discharge; (3) in-hospital mortality, defined as all-cause death during the index AMI hospitalization; and (4) length of hospital stay. Additionally, we examined the change in AMI hospitalizations per 100 000 beneficiary-years by using the total number of AMI hospitalizations for each year divided by the corresponding number of beneficiary-years of fee-for-service enrollments, based on the total months that people were in fee-for-service. We also assessed the Consumer Price Index–adjusted Medicare payment for an index AMI hospitalization, referring to the year 2014. For each year, we also measured the rates of individuals who received catheterization, PCI, and CABG, defined as a patient who underwent a targeted procedure in an acute-care hospital within 30 days of an index AMI admission among admitted patients with AMI in each year.

### Statistical Analysis

We conducted analyses at the patient, hospital, and county levels. At the patient level, we assessed changes in patient characteristics over the study period via a logistic regression to model each individual characteristic as a function of a year parameterized as a count, ranging from 0 to 19, corresponding to years 1995 to 2014. To assess the changes in individual patients’ risk of 30-day deaths over time, we used the 2014 data (the end of the study period data) to fit a mixed model with hospital random effects and a logit link function to model mortality as a function of patient age, sex, and comorbidities. Using the estimated parameters, we calculated an expected probability of 30-day death for each patient hospitalized in 1995 to 2013. We then calculated a standardized mortality ratio as the observed 30-day deaths divided by the expected deaths for each year of 1995 to 2013. For a given year, a ratio greater than 1.0 indicates that the risk of death for such a patient is expected to be lower if that patient were hospitalized in 2014. We conducted a nonparametric bootstrapping with replacement to construct a 95% confidence interval for this standardized mortality ratio. To assess potential inequalities in the changes in patients’ risk of death, we compared the standardized mortality ratios across age, sex, race, and dual-eligible subgroups.

At the hospital level, we assessed hospital performance by calculating a 30-day risk-standardized mortality rate for each hospital and each year based on the CMS method for profiling hospitals.^13,14,17,18^ To assess hospital characteristics associated with mortality declines, we fitted a mixed model with the state as random effects and an identity link function to model the absolute difference in risk-standardized mortality between 1995 and 2014 as a function of hospital characteristics, adjusting for hospitals’ baseline risk-standardized mortality rate.

At the county level, to ensure that each county had an adequate number of patients with AMI, we divided the 20-year period into 5 intervals, each combining 4 sequential years of data (1995-1998, 1999-2002, 2003-2006, 2007-2010, and 2011-2014). We calculated the risk-standardized 30-day mortality rate for each county and each subperiod by extending the CMS method to the county level, adjusted for patient characteristics (age, sex, race [white, black, Hispanic, all others], dual eligibility, and comorbidities). We then mapped counties using a gradient from red, yellow, and green (increase in red, on average in yellow, and decrease in green). To assess county characteristics associated with the decline in mortality, we fitted a mixed model with state as random effects and an identity link function to model the absolute difference in risk-standardized mortality between 1995 to 1998 and 2011 to 2014 as a function of county characteristics. To facilitate data presentation, we report patient characteristics and outcomes by the five 4-year intervals defined in the county-level analysis.

Analyses were conducted between January 15 and June 5, 2018, using SAS statistical software, version 9.4, 64-bit Windows version (SAS Institute, Inc). As of 2019, the data were 5 years old. All statistical tests were 2-tailed, at a significance level of .05.

## Results

### Study Population and Patient Characteristics

There were 4 367 485 unique Medicare fee-for-service patients aged 65 years or older hospitalized for AMI across 5680 unique hospitals during the 20-year period. Between 1995 and 2014, the mean (SD) age of patients with AMI increased from 76.9 (7.2) to 78.2 (8.7) years, an absolute difference of 2.7 years; the proportion of female patients declined from 49.5% to 46.1%; the percentage of white patients declined from 91.0% to 86.2%; and the percentage of black patients increased from 5.9% to 8.0% (all *P* values for trends <.001).

The rate of hospitalization for an initial AMI declined over the period from 914 to 566 per 100 000 beneficiary-years, a relative 38.1% reduction (eFigure 1 in the Supplement). Most comorbidities increased during the study period, but the proportions of patients with cerebrovascular disease, stroke, anterior AMI location, inferior lateral AMI, unstable angina, and valvular heart disease declined (eFigure 2 in the Supplement; Table).

**Table.** Patient Characteristics

Characteristic	1995-1998 (n = 1 012 897)	1999-2002 (n = 999 009)	2003-2006 (n = 919 981)	2007-2010 (n = 742 812)	2011-2014 (n = 692 786)	Aggregated (N = 4 367 485)
Demographic characteristics and comorbidities, No. (%)						
Age, mean (SD), y	77.3 (7.3)	78.4 (7.8)	78.6 (8.1)	78.6 (8.5)	78.1 (8.6)	78.2 (8.0)
Female	505 015 (49.9)	505 786 (50.6)	461 401 (50.2)	366 863 (49.4)	326 029 (47.1)	2 165 094 (49.6)
White	914 985 (90.3)	889 941 (89.1)	809 239 (88.0)	650 931 (87.6)	599 774 (86.6)	3 864 870 (88.5)
Black	61 039 (6.0)	68 416 (6.8)	67 842 (7.4)	56 564 (7.6)	55 536 (8.0)	309 397 (7.1)
Other race	31 440 (3.1)	40 652 (4.1)	42 900 (4.7)	35 317 (4.8)	37 476 (5.4)	193 218 (4.4)
Heart failure	134 488 (13.3)	144 952 (14.5)	136 957 (14.9)	106 029 (14.3)	92 039 (13.3)	614 465 (14.1)
Myocardial infarction	46 391 (4.6)	49 164 (4.9)	45 062 (4.9)	36 292 (4.9)	33 072 (4.8)	209 981 (4.8)
Unstable angina	56 939 (5.6)	49 009 (4.9)	33 820 (3.7)	21 471 (2.9)	17 680 (2.6)	178 919 (4.1)
Chronic atherosclerosis	680 245 (67.2)	655 429 (65.6)	630 149 (68.5)	519 313 (69.9)	496 177 (71.6)	2 981 313 (68.3)
Cardiorespiratory disease	21 737 (2.1)	25 313 (2.5)	27 667 (3.0)	34 521 (4.6)	36 762 (5.3)	146 000 (3.3)
Hypertension	485 258 (47.9)	537 800 (53.8)	532 660 (57.9)	477 564 (64.3)	468 670 (67.7)	2 501 952 (57.3)
Stroke	20 517 (2.0)	20 595 (2.1)	17 841 (1.9)	13 913 (1.9)	12 405 (1.8)	85 271 (2.0)
Cerebrovascular disease	40 588 (4.0)	52 259 (5.2)	42 135 (4.6)	30 768 (4.1)	27 183 (3.9)	192 933 (4.4)
Renal failure	31 353 (3.1)	45 552 (4.6)	64 392 (7.0)	87 220 (11.7)	92 187 (13.3)	320 704 (7.3)
Chronic obstructive pulmonary disease	204 168 (20.2)	227 051 (22.7)	223 856 (24.3)	158 333 (21.3)	138 327 (20.0)	951 735 (21.8)
Pneumonia	91 977 (9.1)	126 982 (12.7)	131 896 (14.3)	114 628 (15.4)	101 380 (14.6)	566 863 (13.0)
Protein calorie malnutrition	9810 (1.0)	24 282 (2.4)	27 512 (3.0)	33 123 (4.5)	36 604 (5.3)	131 331 (3.0)
Dementia	50 051 (4.9)	92 066 (9.2)	95 134 (10.3)	81 219 (10.9)	47 194 (6.8)	365 664 (8.4)
Functional disability	18 090 (1.8)	25 823 (2.6)	21 925 (2.4)	18 419 (2.5)	17 522 (2.5)	101 779 (2.3)
Peripheral vascular disease	52 800 (5.2)	61 473 (6.2)	60 671 (6.6)	49 220 (6.6)	41 585 (6.0)	265 749 (6.1)
Metastatic cancer	23 717 (2.3)	62 611 (6.3)	60 943 (6.6)	50 061 (6.7)	44 158 (6.4)	241 490 (5.5)
Trauma in past year	41 581 (4.1)	51 883 (5.2)	56 273 (6.1)	45 483 (6.1)	37 689 (5.4)	232 909 (5.3)
Major psychiatric disorder	12 524 (1.2)	18 274 (1.8)	16 481 (1.8)	14 918 (2.0)	15 113 (2.2)	77 310 (1.8)
Liver disease	4222 (0.4)	5673 (0.6)	6337 (0.7)	5102 (0.7)	5751 (0.8)	27 085 (0.6)
Depression	22 740 (2.2)	44 332 (4.4)	48 799 (5.3)	40 341 (5.4)	41 069 (5.9)	197 281 (4.5)
Diabetes	334 688 (33.0)	293 433 (29.4)	277 281 (30.1)	225 956 (30.4)	229 613 (33.1)	1 360 971 (31.2)
Outcomes, % (95% CI)						
30-d mortality	19.1 (19.1-19.2)	19.2 (19.2-19.3)	17.1 (17.1-17.2)	15.2 (15.1-15.3)	13.4 (13.3-13.5)	17.2 (17.1-17.2)
In-hospital mortality	14.3 (14.2-14.4)	14.1 (14.0-14.1)	11.5 (11.5-11.6)	9.2 (9.15-9.28)	7.7 (7.63-7.76)	11.7 (11.7-11.8)
30-d readmission	21.3 (21.2-21.4)	21.8 (21.7-21.9)	21.4 (21.3-21.5)	20.2 (20.1-20.3)	17.4 (17.3-17.5)	20.4 (20.3-20.4)
Discharge to home	44.5 (44.4-44.6)	46.1 (46.0-46.2)	45.1 (45.0-45.2)	46.9 (46.8-47.0)	50.2 (50.1-50.3)	46.3 (46.3-46.3)
Discharge to home with home health service	9.1 (9.06-9.17)	9.6 (9.51-9.62)	12.1 (12.1-12.2)	13.2 (13.2-13.3)	13.2 (13.1-13.3)	11.2 (11.2-11.2)
Discharge to skilled nursing home	10.5 (10.4-10.5)	14.0 (13.9-14.1)	15.9 (15.8-16.0)	16.6 (16.6-16.7)	15.4 (15.3-15.5)	11.8 (11.8-11.9)
Discharge to hospice	0 (0.01-0.01)	0.3 (0.30-0.32)	1.7 (1.64-1.69)	2.7 (2.71-2.78)	3.0 (2.96-3.04)	1.4 (1.35-1.38)
Transferred to another acute-care hospital	19.0 (18.9-19.1)	11.7 (11.6-11.7)	8.8 (8.70-8.82)	6.6 (6.57-6.68)	5.6 (5.59-5.70)	10.9 (10.9-11.0)
Length of stay, mean (SD), d	6.5 (6.0)	6.5 (6.7)	6.2 (6.5)	5.7 (5.9)	5.1 (5.2)	6.1 (6.2)

### Changes in Risk of Mortality at the Patient Level

**Figure 1.**
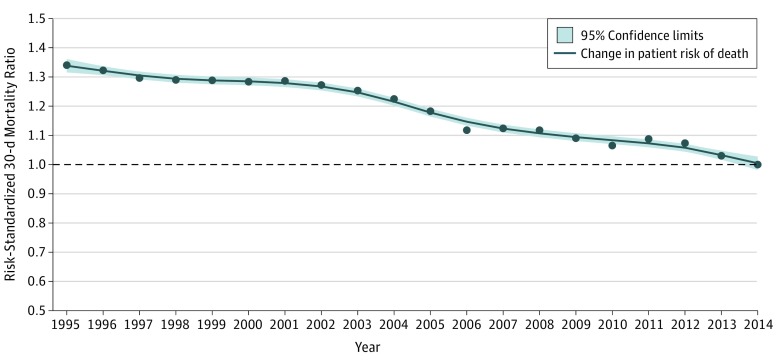
Change in Adjusted Risk of 30-Day Acute Myocardial Infarction Mortality at the Patient Level Between 1995 and 2014 Points indicate year-specific risk-standardized 30-day mortality ratios. The line was smoothed using the locally estimated scatterplot smoothing (LOESS) method (local regression).

Patient risk of 30-day mortality declined significantly over the 20-year period. The observed 30-day mortality declined from 20.0% in 1995 to 12.4% in 2014 (difference, 7.6 percentage points; 95% CI, 7.3-7.8 percentage points). This reduction in observed mortality was found across age, sex, race, and dual-eligibility subgroups (eFigure 3 in the Supplement); the gap in mortality decreased between the dual-eligible and non–dual-eligible subgroups (24.1% vs 19.4%; difference, 4.7 percentage points; 95% CI, 4.3-5.2 percentage points in 1995 and 14.0% vs 12.1%; difference, 1.9 percentage points; 95% CI, 1.4-2.3 percentage points in 2014) and between the female and male subgroups (21.8% vs 18.2%; difference, 3.6 percentage points; 95% CI, 3.4-4.0 percentage points in 1995 and 13.4% vs 11.6%; difference, 1.8 percentage points; 95% CI, 1.4-2.1 percentage points in 2014) (*P* < .001 for interactions). There were no differences in mortality by the race subgroups. Between 1995 and 2014, mortality declined from 13.4% to 7.5% (difference, 5.9 percentage points; 95% CI, 5.6-6.2 percentage points) for patients aged 65 to 74 years, 21.7% to 11.7% (difference, 10.0 percentage points; 95% CI, 9.6-10.4 percentage points) for patients aged 75 to 84 years, and 32.2% to 22.0% (difference, 10.2 percentage points; 95% CI, 9.7-10.9 percentage points) for patients aged 85 years or older. An interaction effect (*P* < .001) between year and age was observed. For a patient hospitalized in 1995, with demographic characteristics and comorbidities unchanged, the adjusted risk of 30-day mortality would be 34% (95% CI, 31.0%-37.2%) lower if the hospitalization occurred in 2014 (Figure 1). This reduction in the adjusted risk of mortality between 1995 and 2014 was lower in patients aged 85 years or older (24%; 95% CI, 20.1%-28.0%), men (25.8%; 95% CI, 21.4%-28.6%), nonwhite and nonblack patients (27.3%; 95% CI, 23.4%-30.5%), and non–dual-eligible patients (32.4%; 95% CI, 29.3%-35.6%) than the national average (eFigure 4 in the Supplement).

### Changes in Risk of Mortality at the Hospital Level

**Figure 2.**
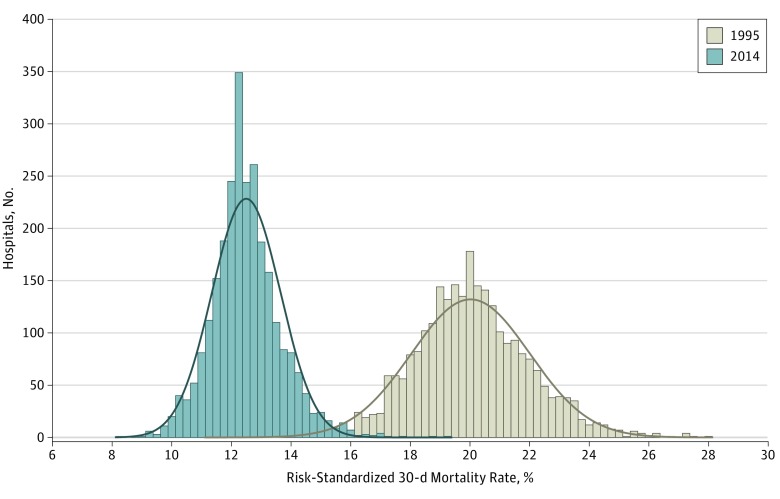
Change in Hospital-Specific Risk-Standardized 30-Day Acute Myocardial Infarction Mortality Rates Between 1995 and 2014 The size of each bar reflects the number of hospitals that filled in a particular interval of risk-standardized mortality rate as well as the distributions (ranges) of rates in 1995 and 2014. The curves represent distribution of the data. Mean (SD) risk-standardized 30-day mortality rates were 20.0% (2.0%) and 12.5% (1.2%) for 1995 and 2014, respectively (n = 2635 hospitals). Restricted to hospitals that had at least 1 case in both 1995 and 2014.

Between 1995 and 2014, the number of unique hospitals that treated at least 1 patient with AMI each year declined from 4989 to 3056; the median (interquartile range) hospital AMI volume remained unchanged from 31 (11-71) to 31 (8-79). The mean (SD) risk-standardized mortality rate, at the individual hospital level, declined from 20.0% (1.7%) in 1995 to 12.5% (1.1%) in 2014 (difference, 7.5 percentage points; 95% CI, 7.4-7.6 percentage points) (Figure 2). The distributions of risk-standardized mortality are shown in eFigure 5 in the Supplement. Overall, the annual national decline was 2.2%.

There were 2635 hospitals represented in both 1995 and 2014. Among these, the absolute change in mortality between 1995 and 2014 ranged from a decrease of 15.4 percentage points to an increase of 2.5 percentage points, with 4 hospitals having increased mortality (increases ranging from 0.7-2.5 percentage points). A reduction in between-hospital heterogeneity in mortality was also observed: the hospital-specific mortality ranged from 11.3% to 28.0% (difference, 16.7 percentage points) in 1995 and ranged from 8.3% to 19.4% (difference, 11.1 percentage points) in 2014. Hospital characteristics were not associated with the decline in risk-standardized 30-day mortality (eFigure 6 in the Supplement).

### Changes in Risk of Mortality at the County Level

**Figure 3.**
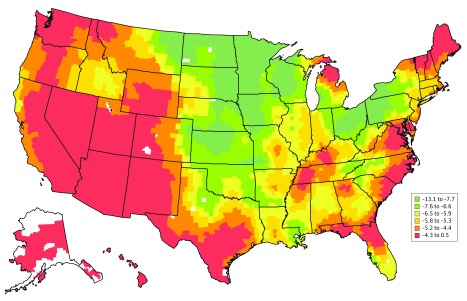
Difference in County-Specific 30-Day Risk-Standardized Acute Myocardial Infarction Mortality Rates Between 1995 to 1998 and 2011 to 2014 The difference was calculated as the percentage point between the 2011 to 2014 combined rate minus the 1995 to 1998 combined rate. Counties are shaded according to the difference in percentage points of the risk-standardized acute myocardial infarction mortality rates (percentage). Counties with negative values had mortality rates that were lower in 2011 to 2014 than in 1995 to 1998. Counties with the largest decline between 2011 to 2014 and 1995 to 1998 are shaded green, while those with the smallest decline or even increase in mortality between 2011 to 2014 and 1995 to 1998 are shaded red. Counties are shaded white if there were missing or insufficient data that precluded the calculation of mortality rates.

From 1995 to 1998 and 2011 to 2014, the county-specific mean (SD) 30-day risk-standardized mortality rate, adjusted for patient characteristics (age, sex, race [white, black, Hispanic, and all others], dual-eligible status, and comorbidities), declined from 19.2% (1.3%) to 13.4% (0.6%). Across counties, the absolute decline in percentage points ranged from 0.1 to 13.1, with 2 counties having increases (0.2 and 0.5 percentage points) (Figure 3). A reduction in between-county heterogeneity in mortality was also observed: the county-specific mortality ranged from 15.4% to 23.1% in 1995 to 1998 and from 12.2% to 13.9% in 2011 to 2014 (eFigure 7 in the Supplement).

Overall, counties with a high rate of individuals enrolled in a Medicare Advantage plan, a high proportion of rural areas, and a high rate of residents who lived in the county for at least 1 year were more likely to have declines in mortality, but counties with high proportions of Medicare beneficiaries not enrolled in the Medicare Part B plan, unemployed residents, and a high risk of potential life lost were less likely to show declines in mortality (eFigure 8 in the Supplement). Counties in the health priority areas were less likely to have a decline in mortality (point estimate = −0.41; 95% CI, −0.60 to −0.23 points).

### Changes in Readmissions, In-Hospital Mortality, Length of Stay, and Medicare Payment

**Figure 4.**
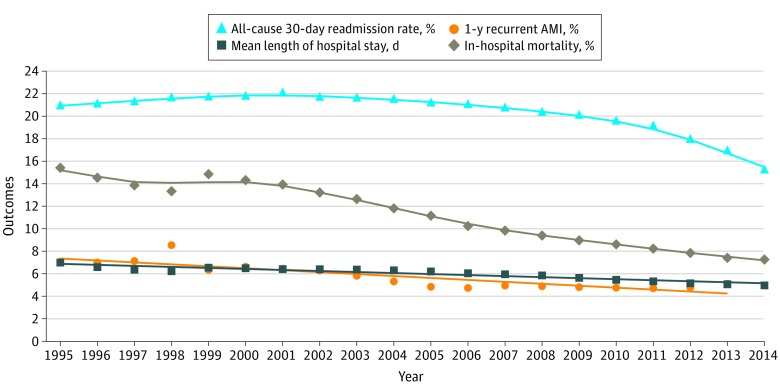
Changes in 30-Day All-Cause Readmissions, 1-Year Recurrent Acute Myocardial Infarction (AMI), In-Hospital Mortality, and Length of Stay Symbols denote observed values and lines represent changes over time. Lines were smoothed using the locally estimated scatterplot smoothing (LOESS) method (local regression). Triangles indicate 30-day all-cause readmissions; circles, 1-year recurrent AMI; diamonds, in-hospital mortality; and squares, length of stay.

Between 1995 and 2014 (2013 for 1-year recurrent AMI), the rates of 30-day all-cause readmissions, 1-year recurrent AMI, and in-hospital mortality declined from 21.0% to 15.3% (difference, 5.7 percentage points; 95% CI, 5.4-6.0 percentage points), 7.1% to 5.1% (difference, 2.0 percentage points; 95% CI, 1.8-2.2 percentage points), and 15.4% to 7.3% (difference, 8.1 percentage points; 95% CI, 7.9-8.3 percentage points), respectively (Figure 4). The median (interquartile range) length of stay declined from 6.0 (3.0-8.0) days in 1995 to 3.0 (2.0-6.0) days in 2014. Between 1995 and 2014, the 2014 Consumer Price Index–adjusted median (interquartile range) Medicare inpatient payment per AMI discharge increased from $9282 ($6969-$12 173) to $11 031 ($8099-$16 861). Although the association between 30-day risk-standardized mortality and Medicare inpatient payment was negative over the study period (eFigure 9 in the Supplement), the effect size was small and it declined slightly over time. In 1995, a $10 000 increase in the payment was associated with an 0.5–percentage point decrease in 30-day risk-standardized mortality; in 2014, the same dollar amount was associated with a decrease of 0.3 percentage points.

### Changes in Inpatient Procedures

Between 1995 and 2014, the rate of 30-day inpatient catheterization increased from 44.2% to 59.9% (difference, 15.7 percentage points; 95% CI, 15.4-16.0 percentage points), the rate of inpatient PCI increased from 18.8% to 43.3% (difference, 24.5 percentage points; 95% CI, 24.2-24.7 percentage points), and the rate of CABG decreased from 14.4% to 10.2% (difference, 4.2 percentage points; 95% CI, 3.9-4.3 percentage points) (eFigure 10 in the Supplement). All 3 procedure rates were correlated at the hospital level. The weighted Pearson correlation coefficients were 0.68 (PCI and CABG), 0.91 (PCI and catheterization), and 0.75 (CABG and catheterization). An increase of 1% in the PCI rate was associated with a decrease in mortality of 0.72 percentage points (95% CI, −0.80 to −0.64 percentage points), but the decrease in CABG rate was not associated with changes in mortality rate. Although we measured these procedures within a 30-day period from the index AMI hospitalization, the majority (96.0% of catheterizations, 96.3% of PCI, and 83.0% of CABG) were performed at the index hospitalization.

## Discussion

From 1995 through 2014, the Medicare fee-for-service population experienced a remarkable and progressive improvement in the hospitalization rates for AMI and the 30-day mortality rate after AMI. In addition, the 2.7-year increase in the average age of patients suggests progress in delaying the onset of AMI. Mortality after AMI is at a historic low of 12%, a reduction of 34% since 1995, accounting for changes in patient characteristics. The improvements were consistent across demographic groups defined by age, sex, race, and eligibility for Medicaid. Almost all hospitals improved over this period, although there was some heterogeneity in the improvement over time, and almost every county experienced improvement, although the size of the change varied. The in-hospital mortality, length of stay, 30-day all-cause rehospitalization, and 1-year recurrent AMI rates also declined, while payments and rates of revascularization increased. The decline in in-hospital mortality was unlikely to have been affected by the decline in length of stay, given the decline in 30-day mortality.

This work extends the prior literature in several ways. Previous studies^2,3,4,7,8,10,11,19^ have reported some of these improvements, but not as comprehensively, or over a 20-year period or across the entire United States. As such, this work reveals novel insights about what has been achieved in reducing and mitigating AMI among Medicare beneficiaries. Moreover, even as payments per hospitalization have increased, outcomes improved and total cost declined because the number of hospitalizations was greatly reduced.

There are several potential explanations for our findings, including changes in the treatment of AMI over this period. By the beginning of the period, it was established that aspirin, β-blockers, and rapid reperfusion therapy could improve outcomes for patients with AMI and that statins and risk factor control could reduce risk. However, it became apparent that the translation of the evidence into practice was incomplete. In the early 1990s, the Health Care Financing Administration (now CMS) launched an improvement initiative focused specifically on AMI. Many patients who were ideal for particular clinical strategies were not receiving them.^20,21,22,23^ Moreover, PCI was emerging as a superior method of reperfusion but was used infrequently, in part because of its availability. Over the years, the use of evidence-based strategies improved dramatically. The speed of reperfusion therapy for ST-segment elevation AMI also improved, as did the use of PCI in general.^3,24^ All of this occurred as a result of concerted efforts by CMS, the American College of Cardiology, the American Heart Association, and other national organizations, along with local hospitals and clinicians, to improve population risk and acute care. Lifestyle changes likely also contributed to the change in the rates of AMI.^25^

We identified some county characteristics that were associated with changes over time. Perhaps most disconcerting is that the areas of the country with persistently highest mortality rates, which we previously termed *health priority areas*, also had slower-than-average decline in AMI mortality.^16^ These areas may particularly benefit from future improvement activities.

### Limitations

The study has several limitations. We examined data about individuals in the Medicare fee-for-service population, and the findings may not reflect the changes in the Medicare Advantage population. Moreover, the growing Medicare Advantage population may have implications for trend analyses, but we have risk adjusted the outcomes data. Also, the hospitalization rate has been dropping over time, and there is no indication that the fee-for-service population is getting healthier over time.^26^ Because this is a Medicare population, we cannot be sure about how our results may reflect the experience of younger populations. The lack of laboratory data in our study precluded our ability to include troponin levels in the risk adjustments. The lack of clinical information in claims data precluded our ability to assess the changes in process of care over time, such as door-to-balloon time and the use of medications, which could explain the changes in outcomes. Nevertheless, other studies^9,27^ have shown remarkable improvement in AMI process measures over this period. Additionally, our assessments of comorbidities are dependent on codes and may not accurately reflect the condition of the patients, but the improvements were also present in unadjusted analyses. There may be some concerns that the higher sensitivity of biomarkers used to diagnose AMI may have contributed to the trend. Changing definitions of AMI do make comparisons difficult and likely led to shifting of patients among the categories of acute coronary syndromes. However, this improvement in mortality occurred in a period of a marked reduction in the number of hospitalizations.^28^ Moreover, the changes in mortality were gradual over time and did not show an abrupt change with the introduction of troponins—or level off after a period of adoption. Also, a systematic review of the validity of administrative codes did not find evidence of a secular trend in code sensitivity, although the evidence is limited.^29^ Finally, we also restricted this study to a period in which *International Classification of Diseases, Ninth Revision, Clinical Modification *codes were used so as not to introduce a new coding system into the assessment of the trends. Also, the effect of the expansion of codes during this period on risk determination is unclear, but this seems unlikely to affect the overall findings of this study.

## Conclusions

This study charts the course of AMI among Medicare fee-for-service beneficiaries over a recent 20-year period. Hospitalization rates for AMI declined 38%, and there were improvements in 30-day mortality, readmissions, length of stay, and 1-year recurrent AMI, with only a modest increase in payments. Revascularization rates increased markedly. Thirty-day adjusted mortality, which declined 34%, touched almost every county and hospital in the country. Nevertheless, there was heterogeneity in the performance over time with some hospital and county characteristics associated with the change. Overall, however, we describe 2 decades of marked improvements in outcomes for AMI among the increasingly smaller number of people in the United States who experience it, representing a transition in the impact of this condition.

## Notes

Supplement. **eTable. **County-Specific Characteristics **eFigure 1.** Change in Number of Initial AMI Hospitalizations per 100,000 Beneficiary-Years, 1995-2014 **eFigure 2.** Annual Relative Changes in Patient Characteristics **eFigure 3.** Trend in Observed Mortality by Year and Sub-population **eFigure 4.** Changes in Patient Risk of 30-Day Mortality Between 2014 and 1995 by Sub-population **eFigure 5.** Nationwide Change in Hospital-Specific 30-Day Risk-Standardized Mortality Rate by Selected Years **eFigure 6.** Hospital Characteristics Associated With Change in Difference in Hospital-Specific Risk-Standardized 30-Day Mortality Rates Between 1995 and 2014 **eFigure 7.** Change in County-Specific Risk-Standardized 30-Day Mortality in 1995-1998 (Top Panel) and in 2011-2014 (Bottom Panel) **eFigure 8.** County-Specific Characteristics Associated With Difference in County-Specific Risk-Standardized 30-Day Mortality Between 1995-1998 and 2011-2014 **eFigure 9.** Association Between Medicare Inpatient Payment and 30-Day Risk-Standardized Mortality at the Hospital Level **eFigure 10.** Annual Changes in the 30-Day Rates of Three Common AMI-Specific Procedures (Catheterization, CABG, and PCI)
