# Supplementary material for: Twenty-Year Trends in Outcomes for Older Adults With Acute Myocardial Infarction in the United States
Source: JAMA Netw Open. 2019 Mar 15;2(3):e191938. doi: 10.1001/jamanetworkopen.2019.1938 (PMC6484647; doi:10.1001/jamanetworkopen.2019.1938)
Supplement: Supplement. — eTable. County-Specific Characteristics eFigure 1. Change in Number of Initial AMI Hospitalizations per 100,000 Beneficiary-Years, 1995-2014 eFigure 2. Annual Relative Changes in Patient Characteristics eFigure 3. Trend in Observed Mortality by Year and Sub-population eFigure 4. Changes in Patient Risk of 30-Day Mortality Between 2014 and 1995 by Sub-population eFigure 5. Nationwide Change in Hospital-Specific 30-Day Risk-Standardized Mortality Rate by Selected Years eFigure 6. Hospital Characteristics Associated With Change in Difference in Hospital-Specific Risk-Standardized 30-Day Mortality Rates Between 1995 and 2014 eFigure 7. Change in County-Specific Risk-Standardized 30-Day Mortality in 1995-1998 (Top Panel) and in 2011-2014 (Bottom Panel) eFigure 8. County-Specific Characteristics Associated With Difference in County-Specific Risk-Standardized 30-Day Mortality Between 1995-1998 and 2011-2014 eFigure 9. Association Between Medicare Inpatient Payment and 30-Day Risk-Standardized Mortality at the Hospital Level eFigure 10. Annual Changes in the 30-Day Rates of Three Common AMI-Specific Procedures (Catheterization, CABG, and PCI) [file jamanetwopen-2-e191938-s001.pdf]

Supplementary Online Content

Krumholz HM, Normand S-LT, Wang Y. Twenty-year trends in outcomes for older adults with acute myocardial infarction in the United States. *JAMA Netw Open*. 2019;2(3):e191938 doi:10.1001/jamanetworkopen.2019.1938

eTable. County-Specific Characteristics
eFigure 1. Change in Number of Initial AMI Hospitalizations per 100,000 Beneficiary-Years, 1995-2014
eFigure 2. Annual Relative Changes in Patient Characteristics
eFigure 3. Trend in Observed Mortality by Year and Sub-population
eFigure 4. Changes in Patient Risk of 30-Day Mortality Between 2014 and 1995 by Sub-population
eFigure 5. Nationwide Change in Hospital-Specific 30-Day Risk-Standardized Mortality Rate by Selected Years
eFigure 6. Hospital Characteristics Associated With Change in Difference in Hospital-Specific Risk-Standardized 30-Day Mortality Rates Between 1995 and 2014
eFigure 7. Change in County-Specific Risk-Standardized 30-Day Mortality in 1995-1998 (Top Panel) and in 2011-2014 (Bottom Panel)
eFigure 8. County-Specific Characteristics Associated With Difference in County-Specific Risk-Standardized 30-Day Mortality Between 1995-1998 and 2011-2014
eFigure 9. Association Between Medicare Inpatient Payment and 30-Day Risk-Standardized Mortality at the Hospital Level
eFigure 10. Annual Changes in the 30-Day Rates of Three Common AMI-Specific Procedures (Catheterization, CABG, and PCI)

This supplementary material has been provided by the authors to give readers additional information about their work.

© 2019 Krumholz HM et al. *JAMA Network Open.*

eTable. County-specific characteristics.

Proportion of senior population (age ≥65) (%)
Divorced (%)
Residence one year ago (%) - Different house in the U.S. - different county
**Geography**
Rural area residents (%)
In health priority area (yes/no)
**Socioeconomic Status**
Bachelor or higher degree (%)
Consumer Price Index-adjusted median income ($10,000)
Proportion of population under the poverty level (%)
Eligible for both Medicare and Medicaid (dual-eligible) (%)
Gini coefficient, continuous (0 to 1)
Percent of unemployed in 2010 (%)
**General Health**
Age-adjusted prevalence of adult diabetes (%)
At least 1 ambulatory visit to a primary care clinician (%)
Years of potential life lost
Emergency department visit rate (%)
**Availability of Care**
Percent of diabetic Medicare beneficiaries receiving HbA1c test (%)
Percent of female Medicare beneficiaries having at least 1 mammogram in 2 yrs (age 67-69) (%)
Enrolled in Medicare Advantage for all 12 months (%)
Not enrolled in supplementary medical insurance (Medicare Part B) (%)
**Health Behaviors**
Age-adjusted rate of physical inactivity (%)

© 2019 Krumholz HM et al. *JAMA Network Open.*

eFigure 1. Change in number of initial AMI hospitalizations per 100,000 beneficiary-years, 1995-2014.

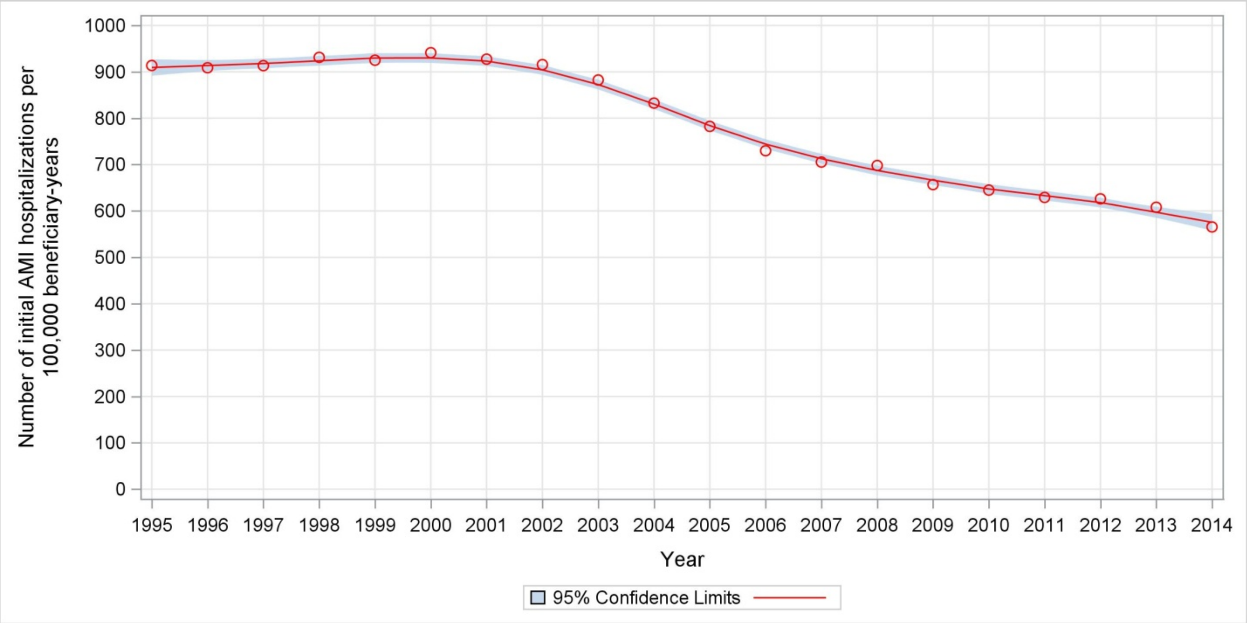

© 2019 Krumholz HM et al. *JAMA Network Open.*

eFigure 2. Annual relative changes in patient characteristics.

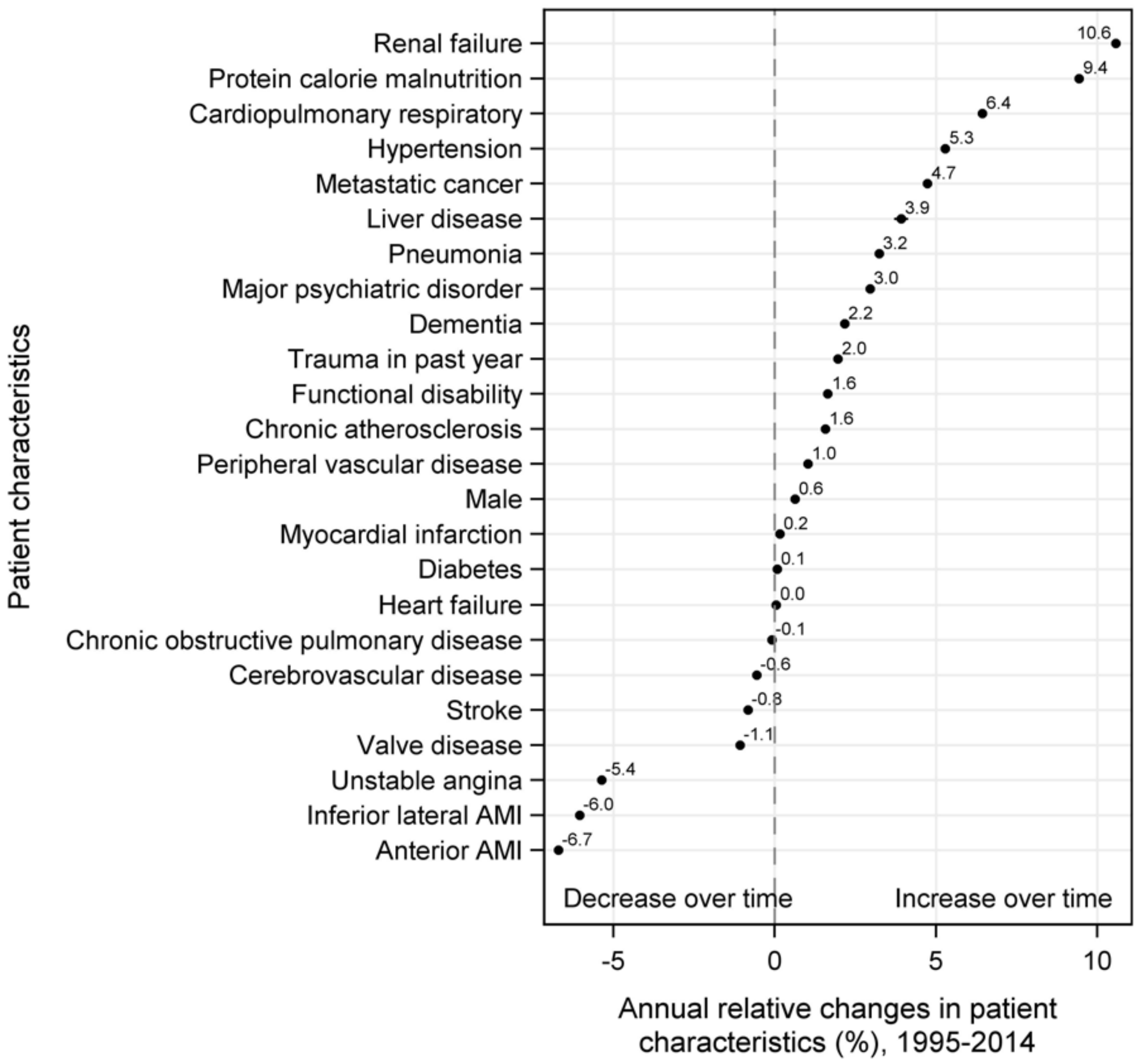

Positive values represent increases in prevalence over time and negative values represent decreases in prevalence over time.

© 2019 Krumholz HM et al. *JAMA Network Open.*

eFigure 3. Trend in observed mortality by year and sub-population.

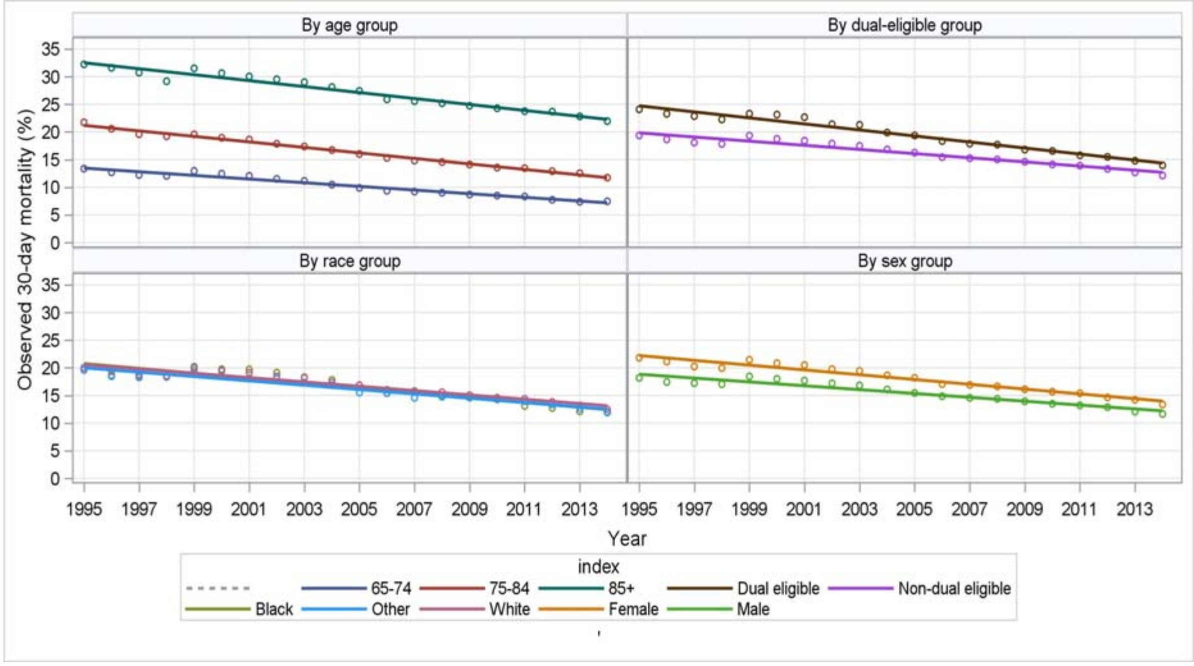

© 2019 Krumholz HM et al. *JAMA Network Open.*

eFigure 4. Changes in patient risk of 30-day mortality between 2014 and 1995 by sub-population.

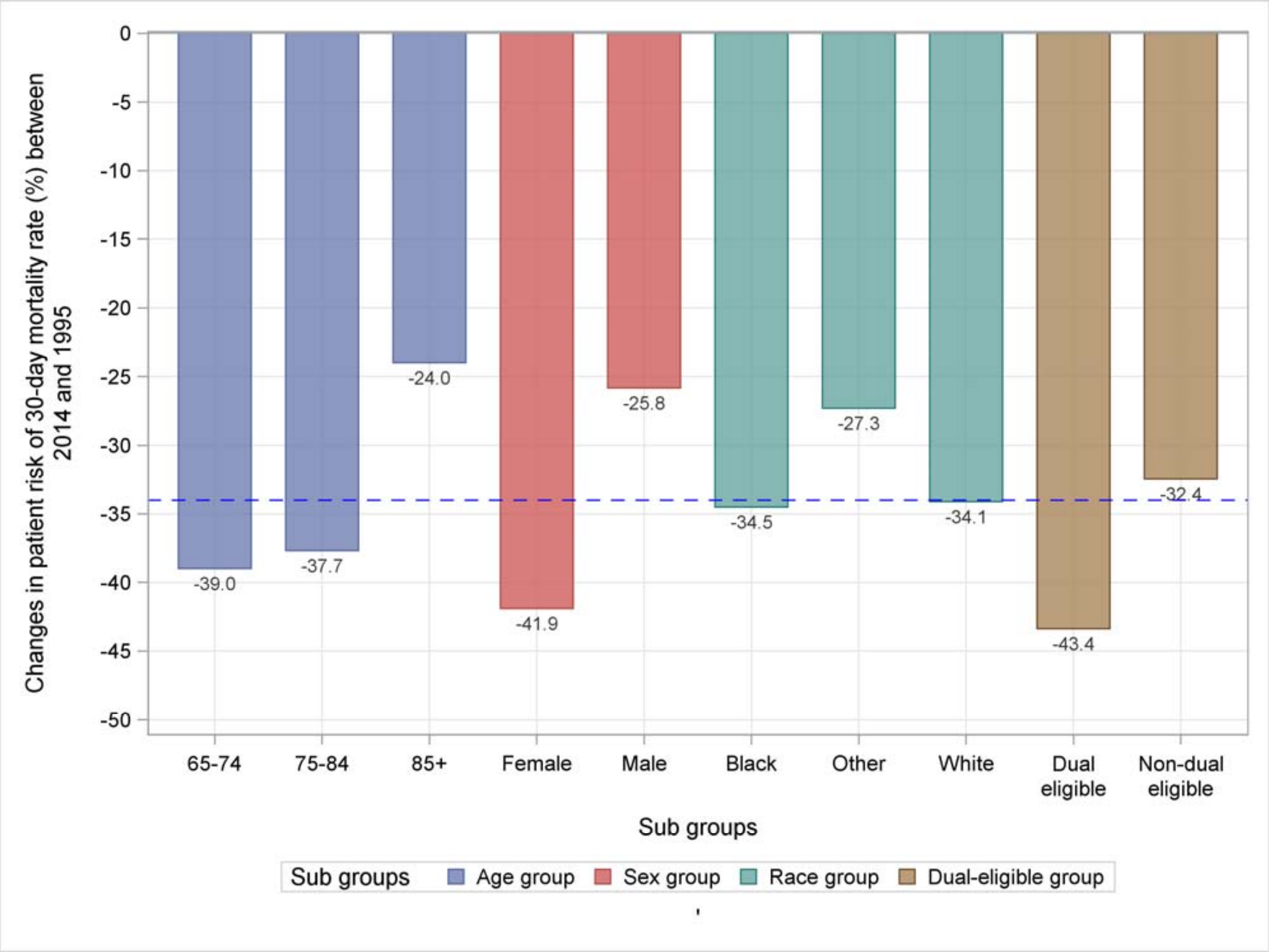

© 2019 Krumholz HM et al. *JAMA Network Open.*

eFigure 5. Nationwide change in hospital-specific 30-day risk-standardized mortality rate by selected years.

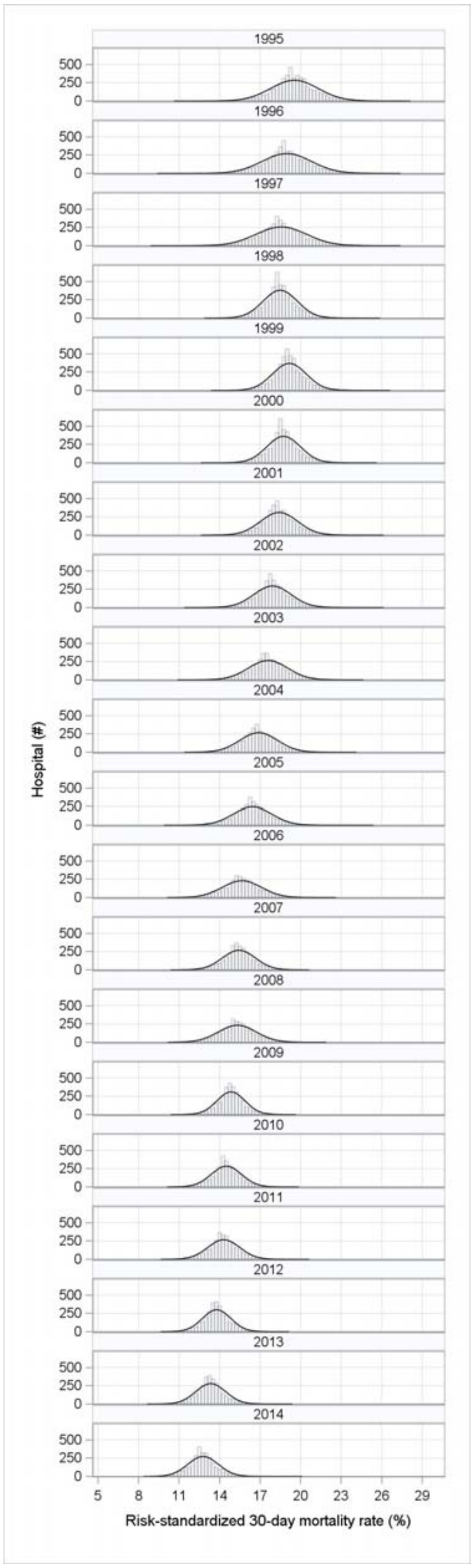

© 2019 Krumholz HM et al. *JAMA Network Open.*

eFigure 6. Hospital characteristics associated with change in difference in hospital-specific risk-standardized 30-day mortality rates between 1995 and 2014.

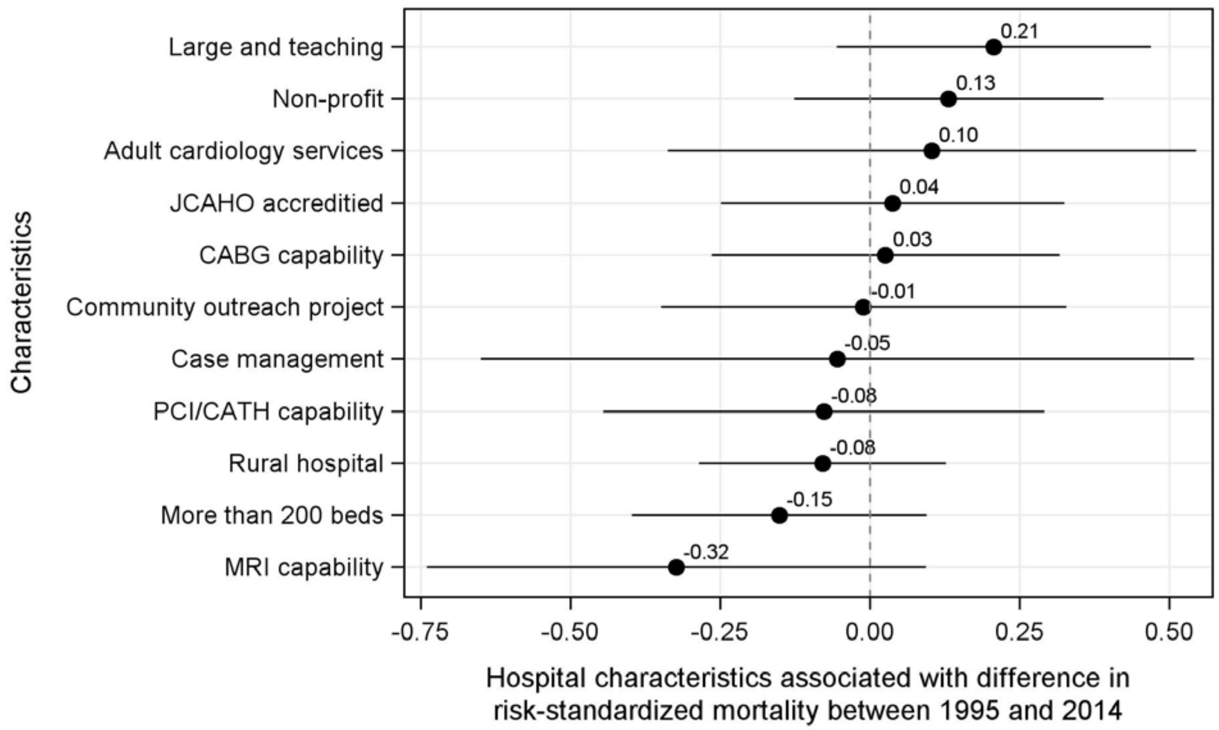

The difference was calculated as the 2014 risk-standardized rate minus the 1995 risk-standardized rate

© 2019 Krumholz HM et al. *JAMA Network Open.*

eFigure 7. Change in county-specific risk-standardized 30-day mortality in 1995-1998 (top panel) and in 2011-2014 (bottom panel). Note: The mortality rate in the highest regions in 2011-2014 was lower than the mortality rate in the lowest regions in 1995-1998.

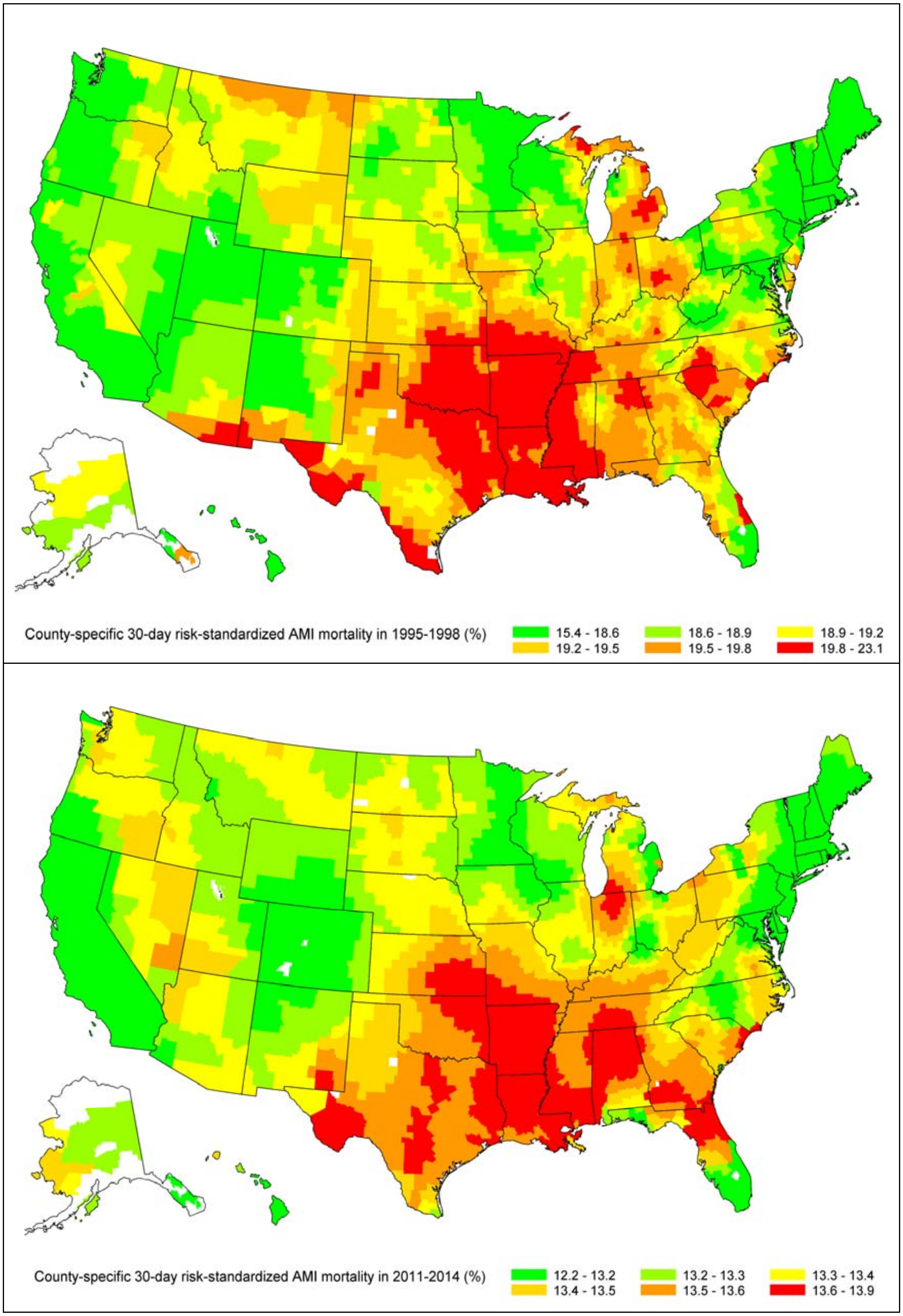

© 2019 Krumholz HM et al. *JAMA Network Open.*

eFigure 8. County-specific characteristics associated with difference in county-specific risk-standardized 30-day mortality between 1995-1998 and 2011-2014.

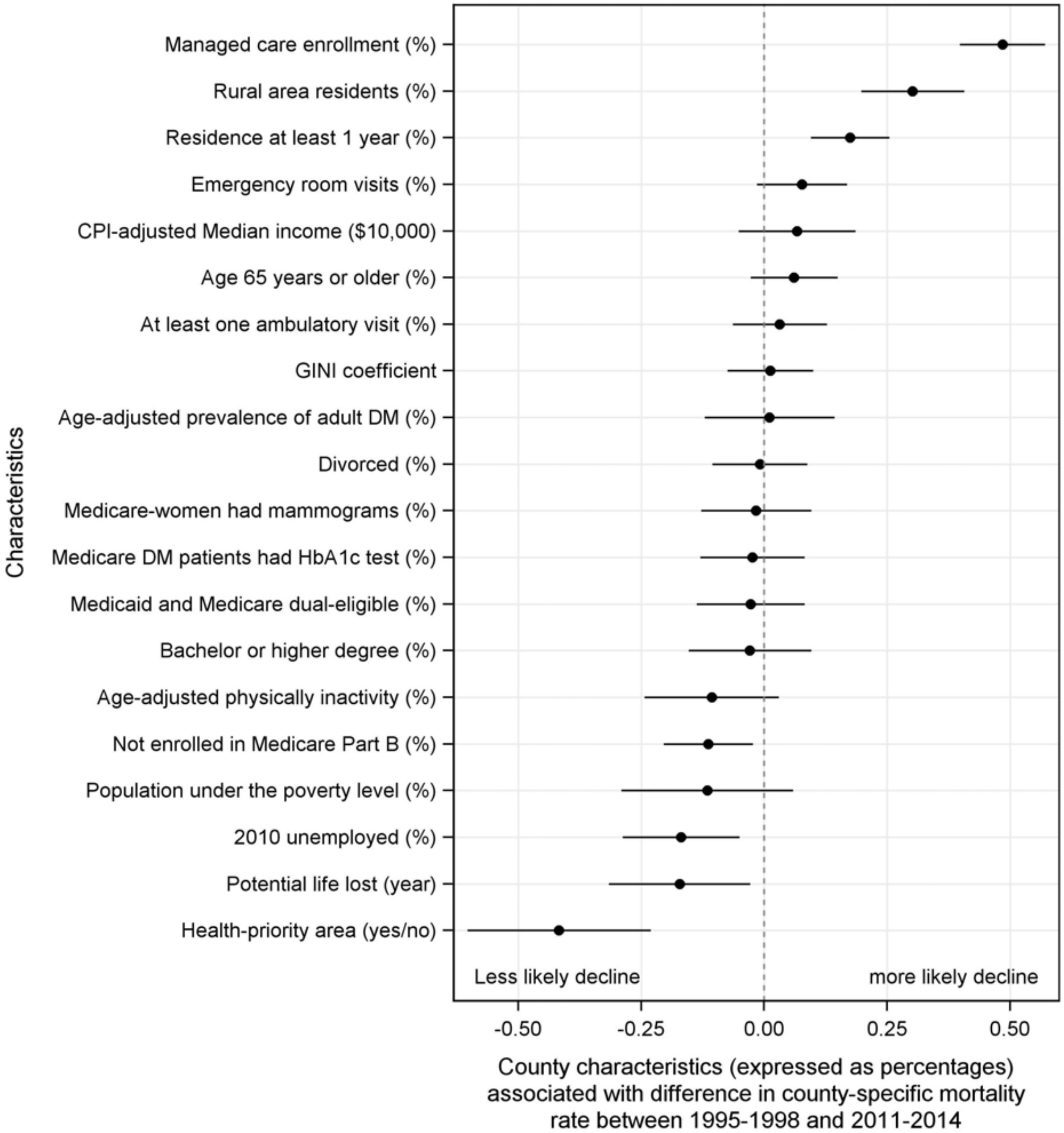

The difference was calculated as the 1995-1998 risk-standardized rate minus the 2011-2014 risk-standardized rate

© 2019 Krumholz HM et al. *JAMA Network Open.*

eFigure 9. Association between Medicare inpatient payment and 30-day risk-standardized mortality at the hospital level.

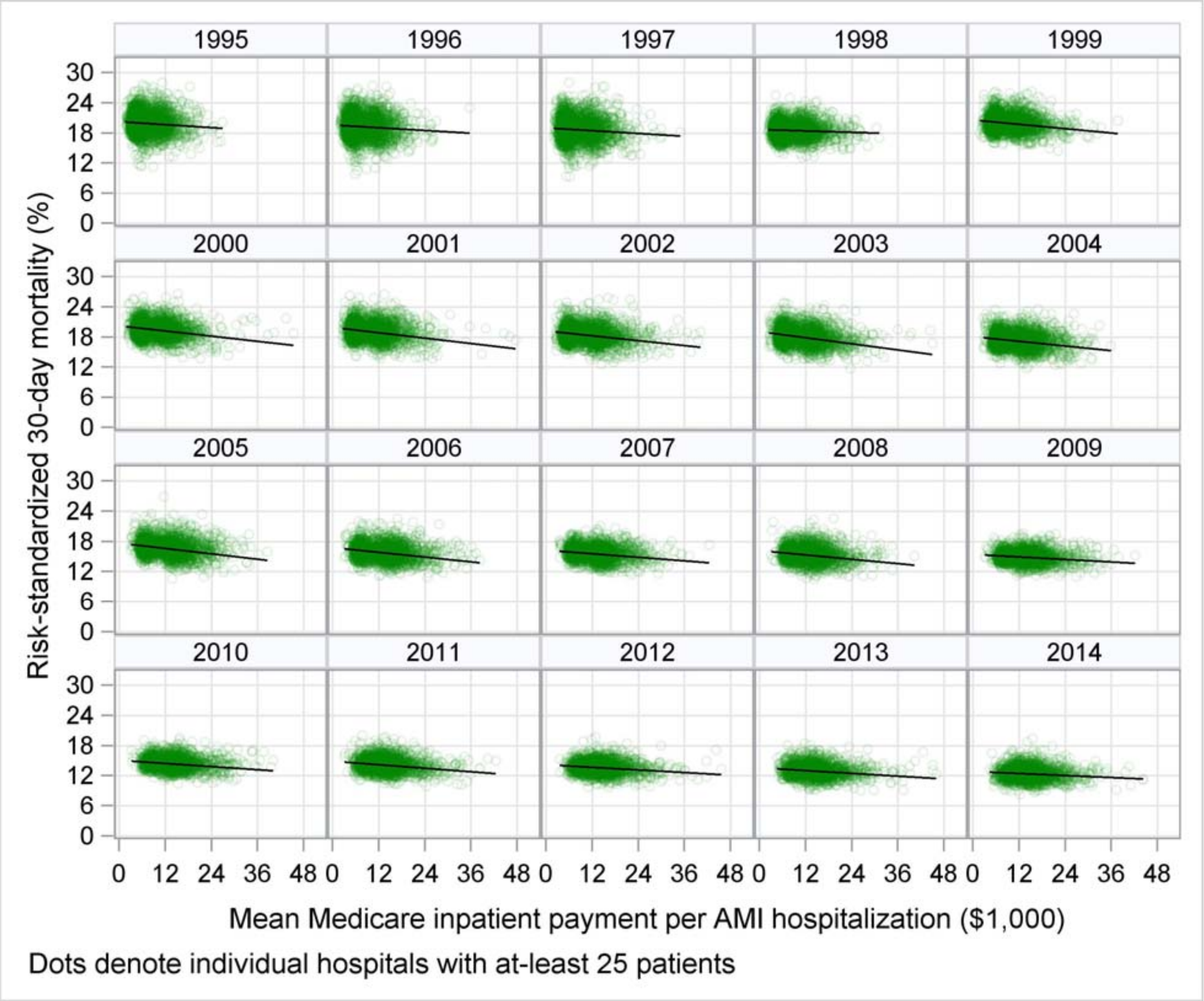

© 2019 Krumholz HM et al. *JAMA Network Open.*

eFigure 10. Annual changes in the 30-day rates of three common AMI-specific procedures (catheterization, CABG, and PCI).

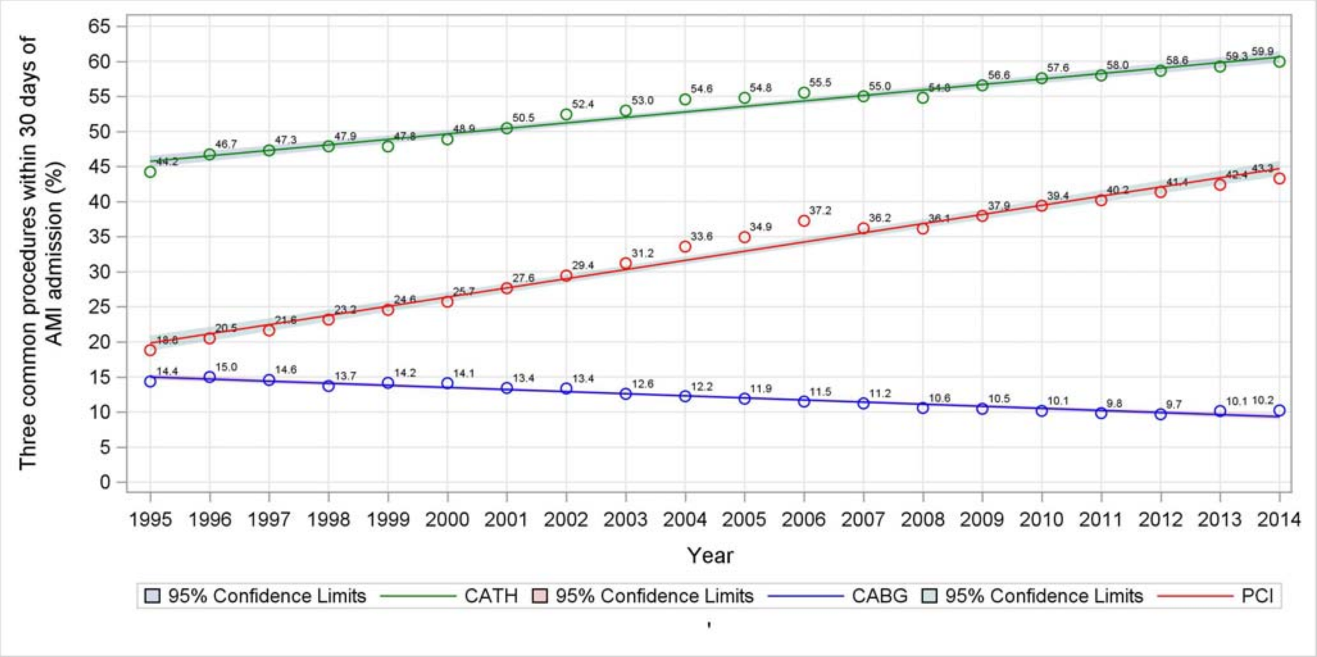

© 2019 Krumholz HM et al. *JAMA Network Open.*
